# Supplementary material for: Dedifferentiated early postnatal lung myofibroblasts redifferentiate in adult disease
Source: Front Cell Dev Biol. 2024 Mar 20;12:1335061. doi: 10.3389/fcell.2024.1335061 (PMC10987733; doi:10.3389/fcell.2024.1335061)
Supplement: Supplementary file 1 [file DataSheet1.PDF]

Supplementary Material

Dedifferentiated early postnatal lung myofibroblasts redifferentiate in adult disease.

Rachana R. Chandran[1,2,5]*, Taylor Adams[3], Inamul Kabir[1,2], Eunate Gallardo[1,2],

Naftali Kaminski[3], Brigitte Gomperts[4,5,6,7,8] and Daniel M. Greif [1,2]*

*** Correspondence:**

rradhamanichandran@mednet.ucla.edu, 847-532-2292 (phone)

daniel.greif@yale.edu, 650-804-4128 (phone), 203-737-6118 (FAX)

1 Supplementary Figures

2 Supplementary Legends

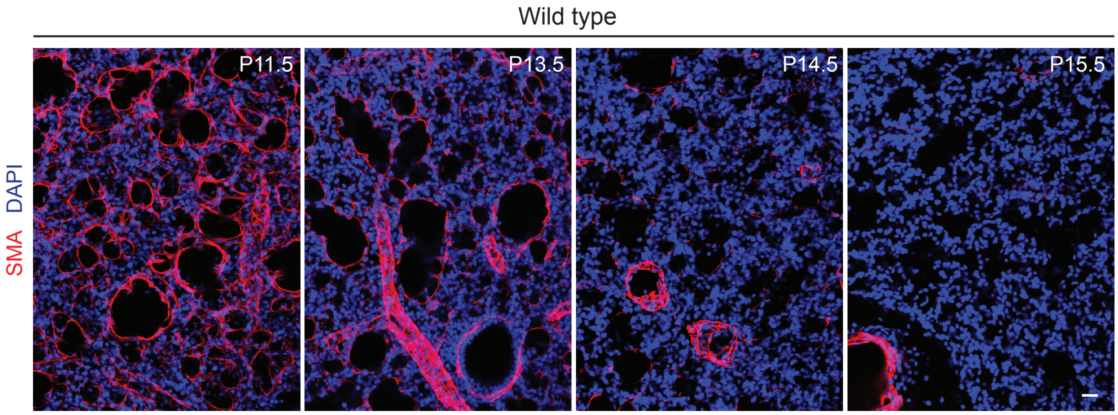
Wild type
SMA DAPI
P11.5
P13.5
P14.5
P15.5

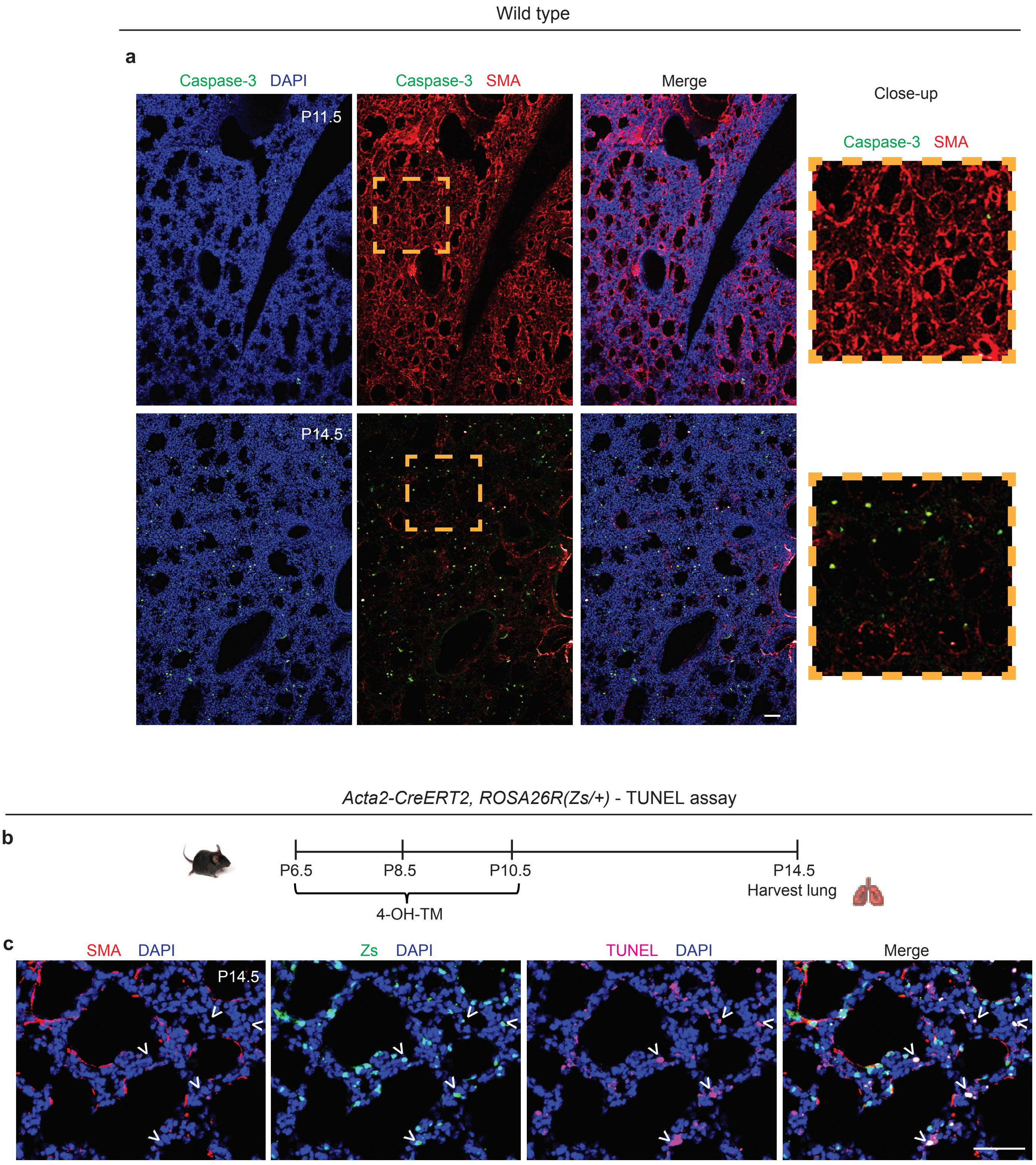
Wild type
a
Caspase-3 DAPI
Caspase-3 SMA
Merge
Close-up
Caspase-3 SMA
P11.5
P14.5
Acta2-CreERT2, ROSA26R(Zs/+) - TUNEL assay
b
P6.5
P8.5
P10.5
P14.5
Harvest lung
4-OH-TM
c
SMA DAPI
Zs DAPI
TUNEL DAPI
Merge
P14.5

Acta2-CreERT2, ROSA26R(Zs/+) - 4-OH TM, P6.5 to P10.5; analysis, P17.5

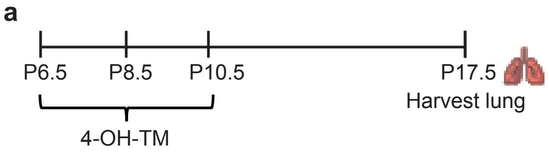

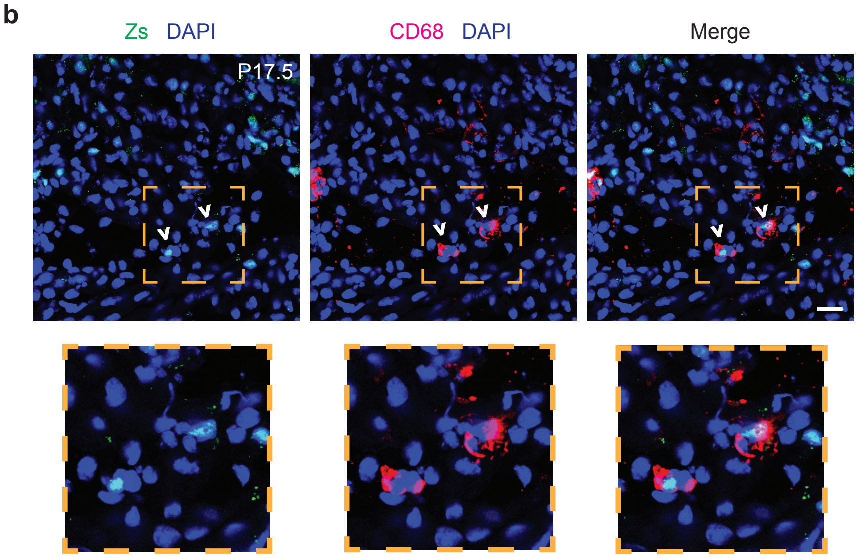

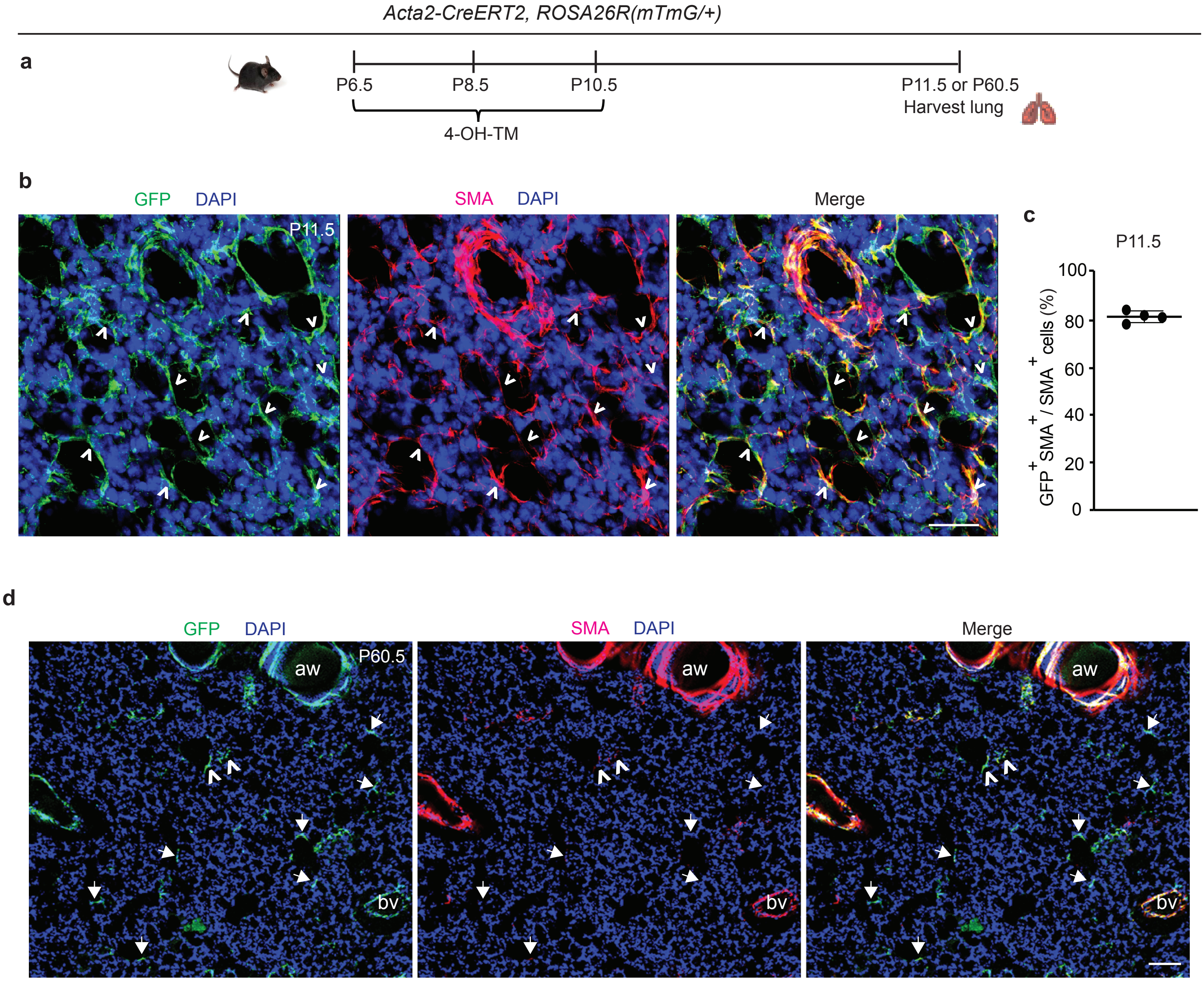
Acta2-CreERT2, ROSA26R(mTmG/+)
a
P6.5
P8.5
P10.5
4-OH-TM
P11.5 or P60.5
Harvest lung
b
GFP DAPI
SMA DAPI
Merge
P11.5
c
P11.5
100
80
60
40
20
0
GFP+ SMA+ / SMA+ cells (%)
d
GFP DAPI
SMA DAPI
Merge
P60.5
aw
bv

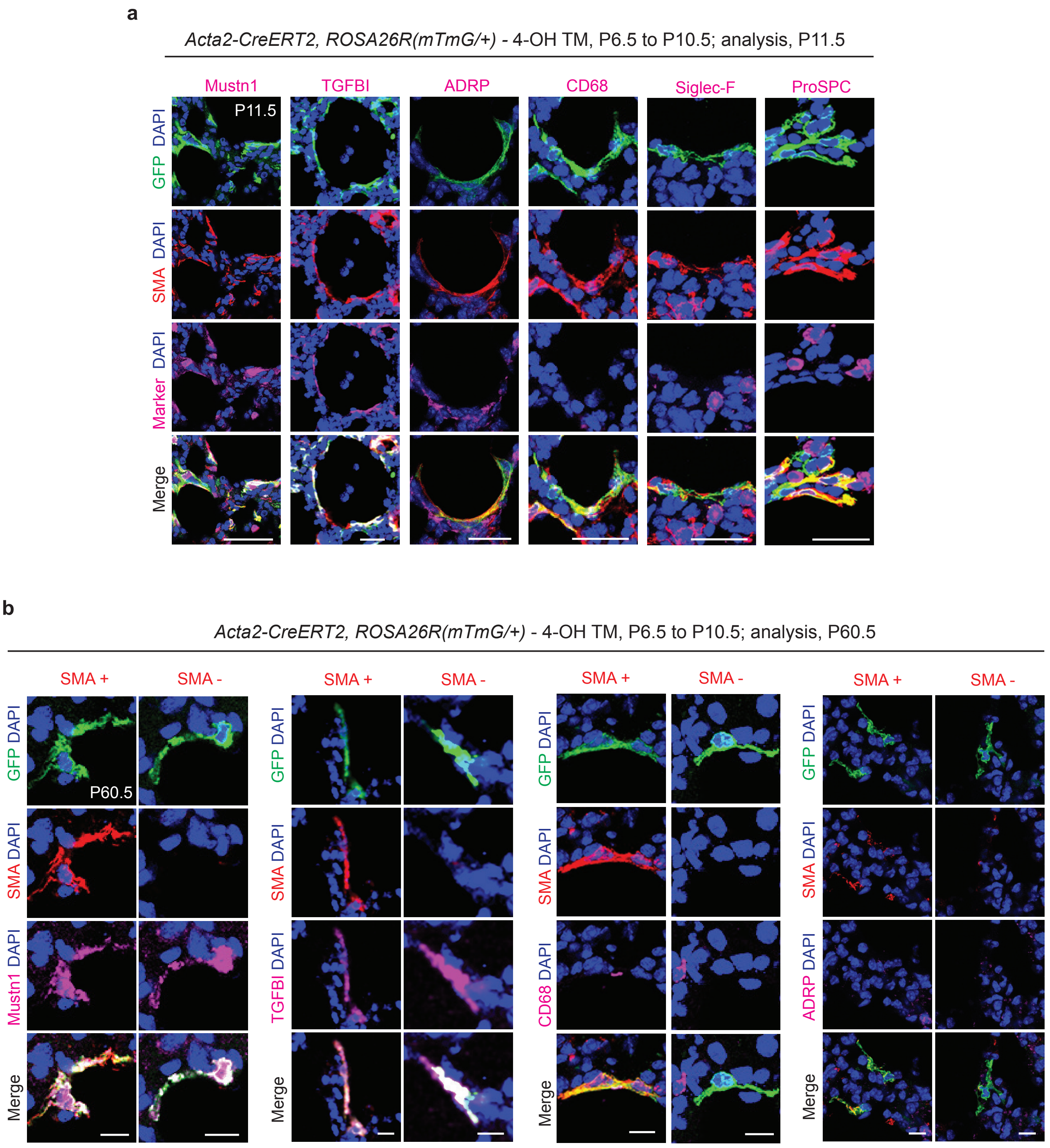
a
Acta2-CreERT2, ROSA26R(mTmG/+) - 4-OH TM, P6.5 to P10.5; analysis, P11.5
Mustn1
TGFBI
ADRP
CD68
Siglec-F
ProSPC
P11.5
GFP DAPI
SMA DAPI
Marker DAPI
Merge
b
Acta2-CreERT2, ROSA26R(mTmG/+) - 4-OH TM, P6.5 to P10.5; analysis, P60.5
SMA +
SMA -
P60.5
GFP DAPI
SMA DAPI
Mustn1 DAPI
TGFBI DAPI
CD68 DAPI
ADRP DAPI
Merge

Acta2-CreERT2, ROSA26R(mTmG/+) - 4-OH TM at P6.5, P8.5 and P10.5; analysis at P60.5

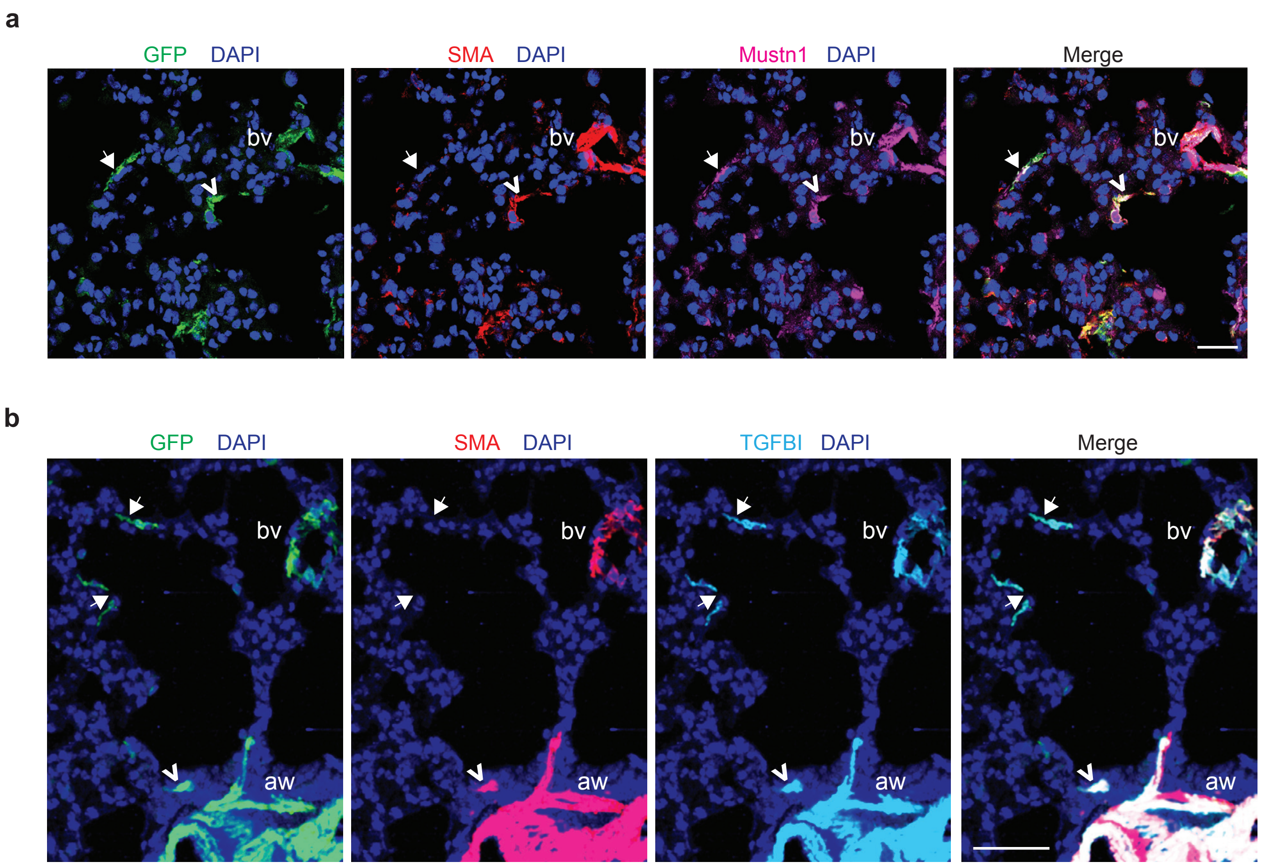

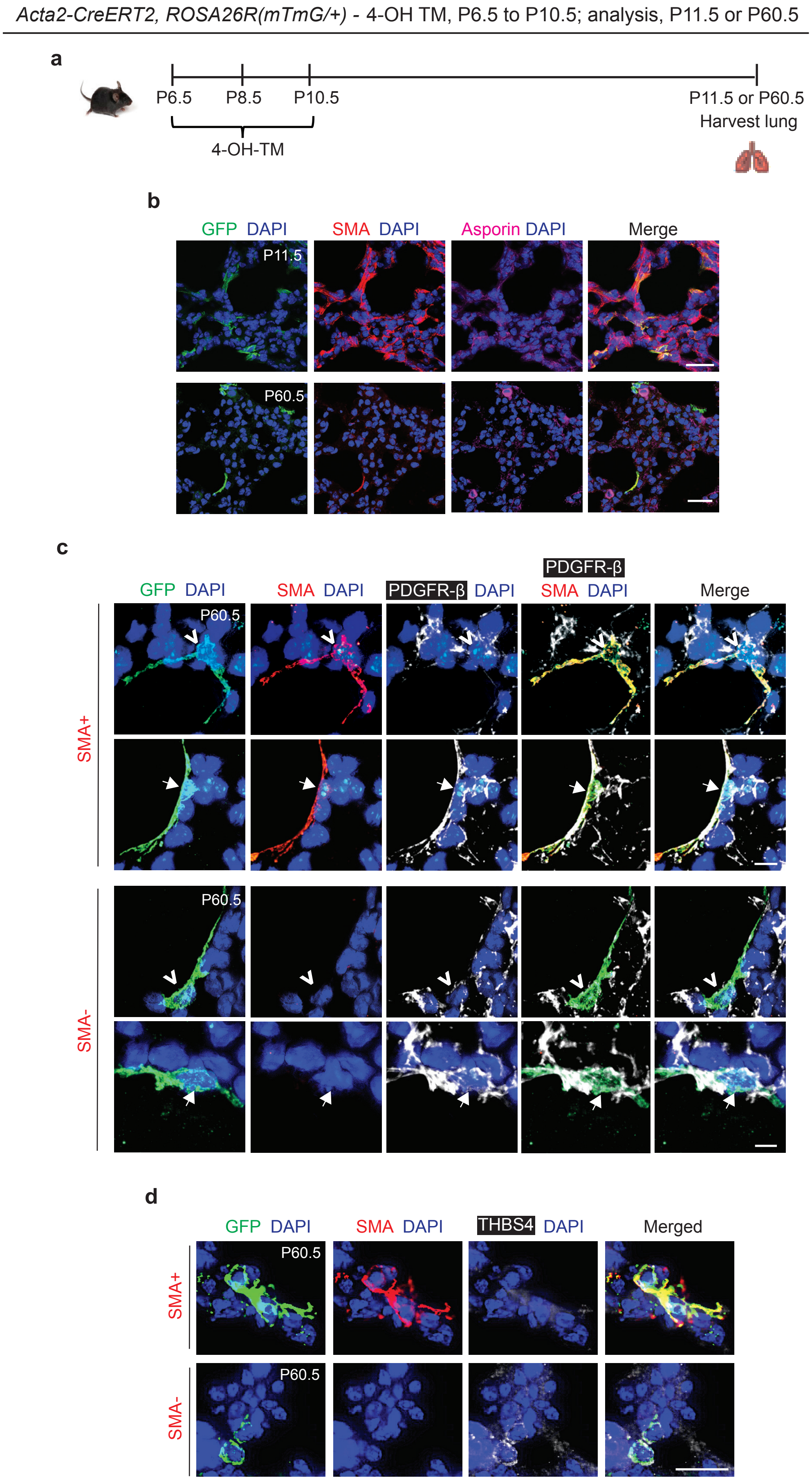
Acta2-CreERT2, ROSA26R(mTmG/+) - 4-OH TM, P6.5 to P10.5; analysis, P11.5 or P60.5
a
P6.5
P8.5
P10.5
P11.5 or P60.5
Harvest lung
4-OH-TM
b
GFP DAPI
SMA DAPI
Asporin DAPI
Merge
P11.5
P60.5
c
GFP DAPI
SMA DAPI
PDGFR-β DAPI
PDGFR-β
SMA DAPI
Merge
SMA+
SMA-
P60.5
P60.5
d
GFP DAPI
SMA DAPI
THBS4 DAPI
Merged
SMA+
SMA-
P60.5
P60.5

Comparison to Tsukui et al. scRNA-seq data

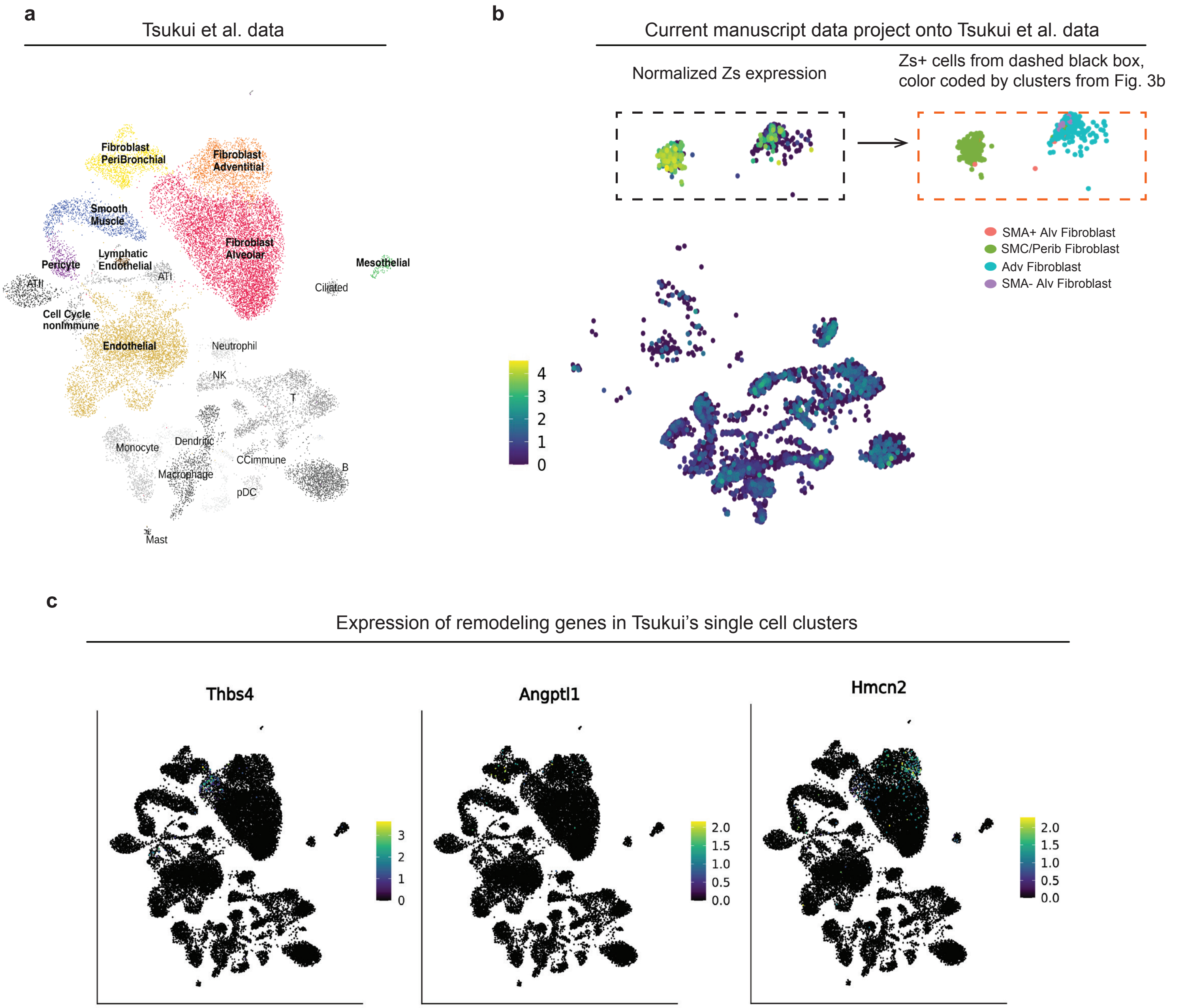

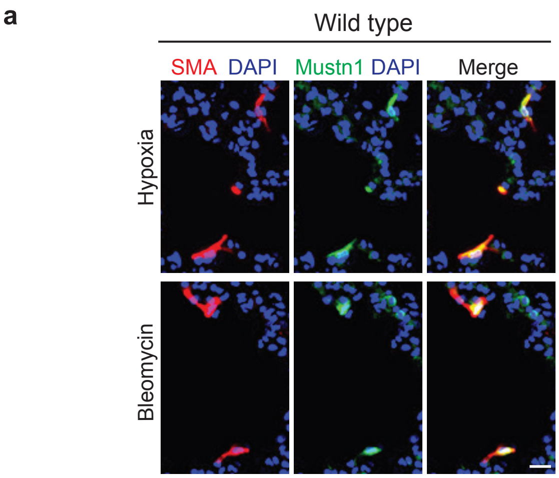
a
Wild type
SMA DAPI
Mustn1 DAPI
Merge
Hypoxia
Bleomycin

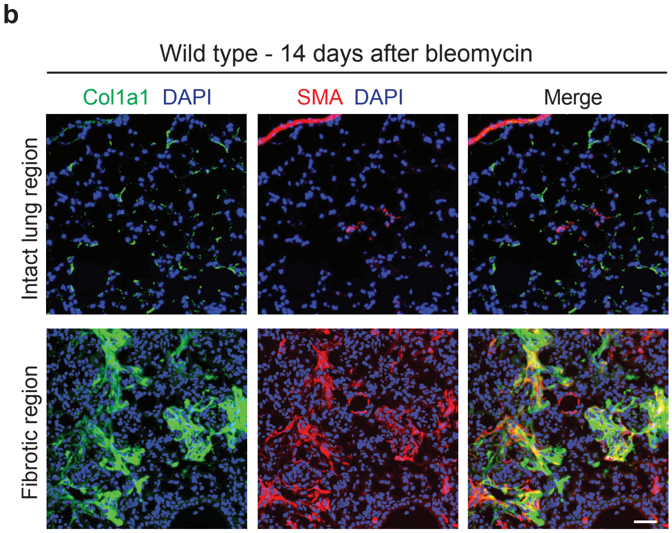
b
Wild type - 14 days after bleomycin
Col1a1 DAPI
SMA DAPI
Merge
Intact lung region
Fibrotic region

Supplementary Figure Legends:

Figure S1. Time course of loss of SMA^+ myofibroblasts in the lung during postnatal alveolarization. Lungs from wild type mice at indicated postnatal ages were harvested, cryosectioned and stained for SMA and nuclei (DAPI). Representative images are shown; n=5 mice per time point. Scale bar, 25 μm.

Figure S2. SMA^+ myofibroblasts downregulate SMA before undergoing apoptosis. a, Cryosections of the lungs of wild type mice at P11.5 and P14.5 stained for caspase 3 and nuclei (DAPI) are shown. Close-ups of boxed regions are displayed on the right; n=3 mice. **b, c,** *Acta2-CreER^{T2}, ROSA26R$^{(Zs/+)}$* mice were induced with 4-OH TM at P6.5, P8.5 and P10.5 and then euthanized at P14.5. Lungs were harvested and cryosectioned. In **b**, schematic of the experiment is shown. In **c**, cryosections were stained for SMA, TUNEL and nuclei (DAPI) and directly imaged for Zs (fate marker); open arrowheads indicate Zs^+TUNEL^+ cells. n=3 mice. Scale bars, 100 μm **(a)**, 50 μm **(c)**.

Figure S3. Association of apoptotic myofibroblasts or their cell debris with macrophages. *Acta2-CreER, ROSA26R$^{(Zs/+)}$* mice induced with 4-OH TM at P6.5, P8.5 and P10.5 were euthanized at P17.5. **a**, Schematic showing the experimental set-up. **b**, Lung cryosections were stained for CD68 (macrophage marker), Zs and nuclei (DAPI). Representative images are shown with close-ups in the boxed region below; open arrowheads indicate co-localization of Zs within $CD68^+$ cells. n=3 mice. Scale bar, 50 μm.

Figure S4. With tamoxifen induction of *Acta2-CreER^{T2}*, *ROSA26R$^{(mTmG/+)}$* mice at P6.5-

P10.5, lung cell marking at P11.5 and P60.5. *Acta2-CreER^{T2}, ROSA26R$^{(mTmG/+)}$* mice were induced with 4-OH TM at P6.5, P8.5 and P10.5. Mice were euthanized at P11.5 or P60.5, and lungs were harvested. **a,** Schematic of the labeling and lung collection timeline is shown. **b**, **d,** Lung sections from mice at P11.5 or P60.5, respectively were stained for GFP (fate marker), nuclei (DAPI) and SMA, and imaged. Open arrowheads indicate GFP$^+$SMA$^+$ cells, and arrows with closed heads indicate GFP$^+$SMA$^-$ cells. Scale bars, 50 µm **(a)**, 100 µm **(b)**. **c**, Labeling efficiency (% of SMA$^+$ cells that are GFP$^+$) was quantified from n=4 mice at P11.5.

Figure S5. Marker analysis at P11.5 and P60.5 of fate mapped early postnatal SMA$^+$ cells. *Acta2-CreER^{T2}*, *ROSA26R$^{(mTmG/+)}$* mice were induced with 4-OH TM at P6.5, P8.5 and P10.5, and lungs were harvested at P11.5 or P60.5. Lung sections were stained for SMA, GFP (fate marker), nuclei (DAPI) and indicated markers (magenta). **a**, Representative images of SMA$^+$GFP$^+$ cells at P11.5 are shown; n=3-6. **b**, Lineage-traced SMA$^+$ and SMA$^-$ parenchymal cells at P60.5 are shown; n=4-6. Scale bars, 25 µm **(a)**, 10 µm **(b)**.

Figure S6. In fate mapped early postnatal SMA$^+$ cells, mustin 1 and TGFBI are expressed in SMA$^+$ and SMA$^-$ cells and in SMCs at P60.5. *Acta2-CreER^{T2}*, *ROSA26R$^{(mTmG/+)}$* mice were induced with 4-OH TM at P6.5, P8.5 and P10.5, and lungs were harvested at P60.5. Lung cryosections were stained for SMA, GFP (fate marker), nuclei (DAPI) and either mustin1 (Mustn1) in **a** or TGFBI in **b**. Open arrowheads indicate GFP$^+$SMA$^+$ myofibroblast, and arrows with closed heads indicate GFP$^+$SMA$^-$ dedifferentiated cells. bv, blood vessel. aw, airway. Scale bars, 25 µm (**a**), 50 µm (**b**).

Figure S7. Marker analysis of early postnatal SMA$^+$ cells at P11.5 and P60.5. *Acta2-CreER^{T2}*, *ROSA26R*$^{(mTmG/+)}$ mice were induced with 4-OH TM at P6.5, P8.5 and P10.5, and lungs were harvested at P11.5 or P60.5. Lung sections were stained for SMA, GFP (fate marker), nuclei (DAPI) and indicated markers (magenta or white). **a,** Schematic of experimental set up is shown. **b,** Staining for asporin as indicated; n=3. Scale bars, 25 µm. **c,** Staining for PDGFR-β as indicated. Representative images of SMA$^+$ and SMA$^-$ cells are shown. Open arrowheads indicate PDGFR-β low cells, and arrows indicate PDGFR-β high cells; n=3 mice. Scale bars, 10 µm. **d**, Staining for Thrombospondin 4 (THSB4) as shown by representative images of SMA$^+$ and SMA$^-$ cells; n=3. Scale bars, 20 µm.

Figure S8. Comparison of scRNA-seq from Figure 3 and a published scRNA-seq dataset. **a,** UMAP representing scRNA-seq of 25,936 cells from 8 murine lungs in Tsukui et al. (*Nat Commun*, 2020, 11:1920). Cell type labels are projected over cell communities. **b,** UMAP of 7,490 cells from our scRNA-seq of an *Acta2-CreER^{T2}, ROSA26R$^{(Zs/+)}$* mouse lung from Figure 3, projected into the UMAP space of Tsukui et al. with normalized ZsGreen expression per cell shown on the left panel. Dashed black box outlines ZsGreen$^+$ cells in clusters used for downstream analysis in our study. In the right panel, these ZsGreen$^+$ cells used for downstream analysis are color coded by clusters from Figure 3b. **c,** UMAPs of Tsukui et al. data showing normalized expression levels per cells for indicated genes.

Figure S9. Co-staining of SMA and mustin1 or collagen 1a1 in lungs of mice exposed to hypoxia or bleomycin. Wild type mice were exposed to hypoxia (FiO_2 10%) for 21 days or to a single dose of bleomycin and then analyzed 14 days later. Lung cryosections were stained

for SMA, indicated markers and nuclei (DAPI); n=3 mice. **a,** Staining for mustin1 with hypoxia and bleomycin exposure. Open arrowheads indicate SMA$^+$ myofibroblasts. **b,** Staining for collagen 1 after bleomycin in fibrotic or intact lung regions. Scale bars, 50 μm
